# A Systematic Overview of Reviews for Complementary and Alternative Therapies in the Treatment of the Fibromyalgia Syndrome

**DOI:** 10.1155/2015/610615

**Published:** 2015-07-13

**Authors:** Romy Lauche, Holger Cramer, Winfried Häuser, Gustav Dobos, Jost Langhorst

**Affiliations:** ^1^Department of Internal and Integrative Medicine, Kliniken Essen-Mitte, Faculty of Medicine, University of Duisburg-Essen, 45276 Essen, Germany; ^2^Department of Internal Medicine 1, Klinikum Saarbrücken, 66119 Saarbrücken, Germany; ^3^Department of Psychosomatic Medicine and Psychotherapy, Munich University of Technology (TUM), 81865 München, Germany

## Abstract

*Objectives.* This systematic overview of reviews aimed to summarize evidence and methodological quality from systematic reviews of complementary and alternative medicine (CAM) for the fibromyalgia syndrome (FMS).* Methods.* The PubMed/MEDLINE, Cochrane Library, and Scopus databases were screened from their inception to Sept 2013 to identify systematic reviews and meta-analyses of CAM interventions for FMS. Methodological quality of reviews was rated using the AMSTAR instrument.* Results.* Altogether 25 systematic reviews were found; they investigated the evidence of CAM in general, exercised-based CAM therapies, manipulative therapies, Mind/Body therapies, acupuncture, hydrotherapy, phytotherapy, and homeopathy. Methodological quality of reviews ranged from lowest to highest possible quality. Consistently positive results were found for tai chi, yoga, meditation and mindfulness-based interventions, hypnosis or guided imagery, electromyogram (EMG) biofeedback, and balneotherapy/hydrotherapy. Inconsistent results concerned qigong, acupuncture, chiropractic interventions, electroencephalogram (EEG) biofeedback, and nutritional supplements. Inconclusive results were found for homeopathy and phytotherapy. Major methodological flaws included missing details on data extraction process, included or excluded studies, study details, and adaption of conclusions based on quality assessment.* Conclusions.* Despite a growing body of scientific evidence of CAM therapies for the management of FMS systematic reviews still show methodological flaws limiting definite conclusions about their efficacy and safety.

## 1. Introduction

The fibromyalgia syndrome (FMS) is a chronic condition that is characterized by chronic widespread pain, fatigue, sleep disorders, cognitive disturbances, and physical and psychological distress [1, 2]. Not only did the diagnostic criteria for FMS change over time but also they are heterogeneous according to the classification system used. While a diagnosis according to the earlier criteria of the American College of Rheumatology required the presence of a specific number of tender points, more recent guidelines did not define tender points but focused on the presence of widespread pain locations [1, 2]. It was estimated that between 2.9 and 3.8% of the general population in Europe and the US are affected [3–5], with the majority of patients in clinical settings being female [2].

Many patients with fibromyalgia utilize complementary and alternative therapies in addition to conventional medicine. The most recent German consumers report indicated that almost every FMS patient had used at least one CAM therapy for the management of FMS in the past, with the majority being heat application or thermal baths (67.0%), CAM medications such as homeopathy, dietary supplements, and vitamins (35.2%), some kind of diet (34.6%), tool-based physical therapies such as acupuncture (28.5%), and meditative exercises such as yoga or tai chi (18.4%) [6]. In an internet survey with US American FMS patients CAM treatments were also highly utilized [7]. Contrary to the highly frequent use data on efficacy and safety are sparse. Such data are however necessary to judge its value within the treatment regimen. Without reliable information such therapies that might benefit FMS patients might also not be recognized by decision makers.

In the current German guideline for the treatment of the fibromyalgia only meditative exercise techniques, that is, yoga, tai chi, or qigong, among others, received a strong recommendation followed by acupuncture with an open recommendation. All other complementary therapies were not recommended when used as monotherapies.

Systematic reviews are literature reviews focusing on the synthesis of evidence from clinical research; they are the basis of evidence-based medicine. Systematic reviews are considered at the highest level of medical evidence; only data from systematic reviews will receive 1a-evidence according to the levels of evidence from the Centre of Evidence-Based Medicine in Oxford [8]. However, no systematic review is like the other, and this especially concerns the methodological quality [9].

For FMS numerous reviews on the effects of complementary and alternative therapies have been published, often with contradictory results. This systematic overview of reviews aimed to provide an overview of evidence from systematic reviews of CAM for fibromyalgia and to determine methodological quality of those reviews.

## 2. Materials and Methods

### 2.1. Protocol and Registration

This review was planned and conducted in accordance with the Preferred Reporting Items for Systematic Reviews and Meta-Analyses (PRISMA) guidelines [10]. The protocol was not registered in any database.

### 2.2. Eligibility Criteria

To be eligible for this overview of systematic reviews, reviews were required to meet the following conditions.

*(1) Types of Reviews*. Systematic reviews with or without meta-analysis were eligible. All reviews claiming to be systematic were included, as well as all reviews with a systematic literature search aiming for a comprehensive overview of evidence. Reviews explicitly stating to have chosen only selected trials, for example, by personal preference, were excluded. 

*(2) Types of Participants*. Only reviews for patients with fibromyalgia were included. Reviews for disease classes that include fibromyalgia, for example, rheumatic diseases in general, were not eligible. No restrictions regarding age, gender, conditions duration, or intensity were applied. There were also no restrictions regarding the diagnostic criteria or procedures of trials and reviews, and they were all considered eligible. 

*(3) Types of Interventions*. Reviews on the effects of complementary and alternative (CAM) therapies were included. For the definition of CAM therapies the definition of the US American National Institute of Health (NIH) [11] was followed; this included natural products, mind and body practices, and other health practices such as holistic medical systems. Reviews were included if they focused on a single therapy or on CAM in general. Reviews focusing on therapy classes that might include CAM therapies, for example, nonpharmacological therapies, were not considered eligible. 

*(4) Types of Outcomes*. Reviews were eligible if they assessed at least one of the following patient-centered outcomes, namely, pain, quality of life, sleep quality, fatigue, psychological distress, well-being, and/or safety. Those outcomes were chosen because they reflect the main symptoms and complaints in FMS patients [2]. 

*(5) Accessibility of Data*. Reviews were eligible only if they were published as full papers. Only published work in peer-reviewed journals was included; abstracts or unpublished work was excluded. Only reviews in English or German were eligible.

#### 2.2.1. Literature Search

The following electronic databases were searched from their inception to September 25, 2013: PubMed/MEDLINE, Scopus, and the Cochrane Library. The literature search was constructed around search terms for “fibromyalgia syndrome,” reviews, and meta-analyses and adapted for each database. For example, the following search strategy was used on the PubMed/MEDLINE database:

 (Fibromyalgia [MeSH Terms] OR fibromyalgia [Title/Abstract] OR fibrositis
 [Title/Abstract] OR FMS [Title/Abstract])
 
**AND**
 (Review [Publication type] OR meta-analysis [Publication type] OR systematic [Subset] OR systematic review [Title/Abstract] OR review
 [Title/Abstract] OR meta-analysis [Title/Abstract]).

The reference lists of identified reviews were also searched manually for relevant articles.

#### 2.2.2. Review Selection

At first all duplicates were removed from the references. Two reviewers (RL, HC) then screened the abstracts of the remaining papers individually and went on to obtain the full papers for potentially eligible reviews. The reviews were then checked in detail, with eligible papers being included in this overview.

#### 2.2.3. Data Collection

Two reviewers (RL, HC) independently extracted data on reviews' characteristics (intervention, comparator, types of included studies, number of studies and patients, information on meta-analysis, risk of bias assessments, and safety). Disagreements were checked with a third reviewer (JL) and resolved by agreement.

#### 2.2.4. Methodological Quality

Methodological quality of reviews was determined using the assessment of the methodological quality of systematic reviews (AMSTAR) [12, 13].

The AMSTAR instrument is an 11-item assessment tool mainly for intervention reviews with good validity and reliability [12, 13]. The AMSTAR determines whether most important contents of systematic reviews have been provided, such as an a priori design, a comprehensive literature search, information about study selection and data extraction, a list of included and excluded studies, characteristics of studies, a quality assessment of included studies, an appropriate method of combining findings or forming conclusions, and conflict of interests statements.

## 3. Results

### 3.1. Literature Search

**Figure 1:**
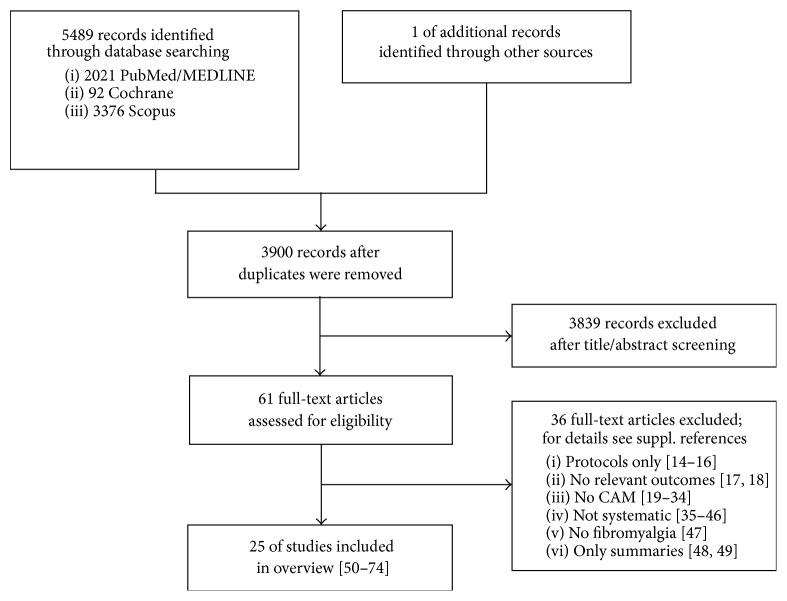
CONSORT flow chart of review inclusion.

The literature search and cross-reference search retrieved 5489 records, of which 1590 were duplicates (Figure 1). After abstract screening, 3839 records were excluded. Of the remaining 61 articles that were assessed as full text, 36 were excluded for the following reasons: three of them were only protocols [14–16], two did not report relevant outcomes [17, 18], 16 did not fit the reviews definition of CAM therapies as stated in the inclusion criteria [19–34], 12 reviews were not systematic [35–46], one did not review trials on fibromyalgia [47], and two publications were only summaries of the results of reviews and medical guidelines [48, 49].

### 3.2. Review Characteristics

Finally 25 reviews could be included; characteristics are presented in Table 1.

The trials were published in the years 2000 (*N* = 1), 2003 (*N* = 1), 2007 (*N* = 1), 2008 (*N* = 1), 2009 (*N* = 5), 2010 (*N* = 4), 2011 (*N* = 2), 2012 (*N* = 2), and 2013 (*N* = 8).

Reviews investigated the following therapies: complementary and alternative therapies in general (*N* = 4) [50–53], exercised-based therapies (*N* = 4) [54–57], manipulative therapies (*N* = 3) [58–60], Mind/Body therapies (*N* = 5) [61–65], acupuncture (*N* = 4) [66–69], balneotherapy (*N* = 3) [70–72], phytotherapy (*N* = 1) [73], and homeopathy (*N* = 1) [74].

RCTs and quasi-RCTs only were included by 17 reviews [50, 51, 53, 55, 56, 58, 62–64, 66–69, 73, 74]; the other reviews also included controlled clinical trials [52, 54, 61, 65], observational trials [70], or any kind of study/review [57, 59, 60]. Reviews included from 4 to 60 trials with a total patient sample from 163 to 2897. Of the 25 reviews only 11 conducted meta-analyses.

All but three reviews [59, 68, 70] assessed the risk of bias of included trials, nine used the Jadad score [51, 54, 57, 58, 63, 64, 69, 73, 74], three used the Cochrane risk of bias assessment [56, 65, 66], three used the van Tulder scale [67, 71, 72], four applied self-adapted instruments or partial instruments [53, 55, 62], one the Oxford rating scale [60], one the Consort rating scale [52] and one the Scale for rating of quality of psychological trials in pain [61]. Only two reviews explicitly reported methods for formulating recommendation [56, 65], and both used the GRADE approach [75].

Quality assessment revealed maximal differences between the reviews; while some were of highest quality, others did only receive 2 of 11 possible points; see Table 2. While all reviews conducted a systematic literature search (which explicitly was an inclusion criterion), some of the reviews did not state an a priori design (*N* = 3) [59, 64, 68], and in some reviews either data extraction was not described or data were not extracted independently by two people (*N* = 12) [50–54, 57, 59, 60, 64, 68]. Only seven reviews listed included and excluded studies [55, 56, 61, 62, 65, 71, 72], and comprehensive study characteristics were provided by 14 reviews only [50, 54–57, 59, 61–63, 65, 67, 71, 73]. Risk of bias assessment was conducted in all but two reviews [59, 68], but the scientific quality was not appropriately included in formulating conclusions in nine of those reviews using a risk of bias assessment [50, 53, 55, 57, 59, 60, 62, 64, 69]. Of those reviews conducting meta-analyses [53, 55–57, 61, 62, 65–68, 71] only one used nonappropriate methods to combine study findings [57].

Likelihood of publication bias was not assessed in the majority of reviews, and two reviews with meta-analysis lacked this information [67, 68]. Conflict of interests was stated in most reviews with only 3 exceptions [52, 63, 72].

### 3.3. Results of Reviews

Evidence for or against the respective interventions is summarized in Table 3.

#### 3.3.1. CAM in General

Altogether four reviews stated that they investigated the effects of CAM in fibromyalgia [50–53], and only one of them conducted a meta-analysis [53]. The first thing that became evident was that no uniform definition of CAM exists; while Holdcraft et al. [52] and Terhorst et al. [53] followed the definition set by the NIH [11], Baranowsky et al. [50] excluded dietary, nutritional, herbal, or hormonal supplements. De Silva et al. [51] on the other hand defined CAM as oral or topical preparations only. Even within the NIH definition results of literature search showed a tremendous increase in studies between 2003 and 2011, resulting in 60 RCTs in 2011 [53] compared to 22 included in 2003 [52]. Quality of CAM reviews was rather low with ranges from 4 to 6 according to the AMSTAR rating.

Most of those reviews presented separate results for single CAM treatments; therefore their results were split up and presented within the respective intervention categories.

#### 3.3.2. Exercise-Based Interventions

Two reviews, with and without meta-analyses, investigated the effects of qigong [54, 56]. While the first review [54] concluded that it was too early to draw conclusions, one year later a second review [56] with almost twice as many studies found moderate-to-strong short-term effects on most patient relevant outcomes when compared to usual care.

Within the more comprehensive reviews results were more equivocal [50, 53, 55, 57]. For yoga as well as tai chi reviews stated mainly positive results [53, 55, 57], even though for tai chi only limited evidence was available [55]. Quality of reviews within this category was mixed with two reviews of high and two of moderate quality.

#### 3.3.3. Manipulative Therapies

Two reviews were conducted for chiropractic management [58, 60] and one was conducted for massage [59], but no meta-analysis was included in either. The two reviews for chiropractic care were of moderate quality, and they found limited evidence [60] or no evidence [58] for effects of chiropractic intervention. Similar results were reported by the comprehensive reviews [52, 53].

The review on massage was judged to be low quality, and its conclusion was that “existing literature provides modest support for use of massage therapy in treating fibromyalgia.” Holdcraft et al. [52] come to a similar conclusion, while Terhorst et al. [53] concluded that there was no evidence for effects of massage at all.

#### 3.3.4. Mind/Body Interventions

Reviews on Mind-Body interventions investigated the effects of any treatment modality [63], meditation-based interventions [64], mindfulness-based stress reduction (MBSR) [65], hypnosis or guided imagery [61], or biofeedback [62]; only 3 of them conducted meta-analyses [61, 62, 65]. Two comprehensive reviews also included Mind/Body interventions [50, 52]. Quality of reviews was considerable heterogeneous with a range from very low [64] to very high [61].

Almost all reviews found at least limited evidence for effects of Mind/Body interventions with the exception of biofeedback [50] and EEG biofeedback in particular [62]. Limited evidence was also found for relaxation [52] but not autogenic training [50].

#### 3.3.5. Acupuncture

Four reviews were found for the effects of acupuncture in FMS [66–69], and three comprehensive reviews included acupuncture [50, 52, 53]. Results for acupuncture were rather varying with reviews finding strong evidence for effects on pain [52, 66, 67], two finding no evidence for effects [53, 68] and two with inconclusive results [50, 69]. Quality of reviews for acupuncture was mixed, with two low and two high quality reviews.

#### 3.3.6. Hydrotherapy/Balneotherapy

For balneotherapy and/or hydrotherapy three reviews were found [70–72]. Together with the comprehensive reviews [50, 52, 53] only positive evidence of different degrees was found [50, 52, 53, 70–72]. Quality again was very different between the reviews from very low to very high quality and only one meta-analysis.

#### 3.3.7. Phytotherapy

For phytotherapy only one review was available [73] and results indicate a need for further studies before any judgment about effects can be made. This review was of moderate quality.

#### 3.3.8. Homeopathy

For homeopathy only one standalone review was available [74], and authors concluded that effectiveness remained unproven. From the more comprehensive reviews one was coming to the same conclusion, while three others found at least some/limited evidence [50–52].

#### 3.3.9. Others

The comprehensive reviews also included nutritional supplements, some of which had limited evidence [52], while others were considered ineffective [53].

### 3.4. Adverse Events

Adverse events were neither assessed nor reported in 14 reviews [50, 52, 54, 58, 64, 68–72, 74]. In the other reviews adverse events were mild to moderate; however as was pointed out before, many trials did not sufficiently report on adverse events.

## 4. Discussion

Altogether 25 systematic reviews investigated the effects of complementary and alternative therapies for FMS. Topics included CAM in general, exercised-based CAM therapies, manipulative therapies, Mind/Body therapies, acupuncture, balneotherapy, phytotherapy, and homeopathy. Two-thirds of the reviews included RCTs and quasi-RCTs; however a substantial number also included other study types from case reports to reviews. Only 11 reviews conducted a meta-analysis. A risk of bias assessment of included trials was included in most reviews; standardized recommendations for or against specific therapies on the other hand were used by two reviews only. Methodological quality of reviews was completely diverse ranging from lowest to highest possible quality.

### 4.1. Homogeneous Findings

Positive results were found for tai chi, yoga, Mind/Body interventions in general, meditation-based interventions, mindfulness-based stress reduction, hypnosis or guided imagery, and balneotherapy or hydrotherapy. Negative results were reported on autogenic training, and inconclusive findings were reported for phytotherapy, however only one review each was available for autogenic training and phytotherapy.

### 4.2. Heterogeneous Findings

Heterogeneous findings, that is, positive and negative findings, were reported for qigong, chiropractic interventions, biofeedback, acupuncture, and nutritional supplements. In the following we will try to determine the source of heterogeneity.

For qigong three reviews found positive evidence [53, 56, 57], and two found negative evidence [50, 55]. Positive reviews were associated with later publication date, higher methodological quality, and three times as many included trials as negative reviews. Therefore positive results appear to be more valid than negative results.

For chiropractic interventions there is one review with limited evidence [60] versus two reviews that do not find positive evidence [52, 58]. No association between time of publication, methodological quality, and number of included trials could be found. However the positive review included all study types limiting the validity of any conclusion. Altogether for chiropractic interventions there seems to be no reliable positive evidence.

For biofeedback two reviews conclude positive results [52, 62] and two do not [50, 62]. One reason might be that only one of the reviews distinguished between EEG and EMG biofeedback [62]. So while for EMG biofeedback positive results have been found, no evidence was present for EEG biofeedback.

For acupuncture the following was found: the later reviews were published, the more trials were included, the more frequent meta-analyses were conducted, and the more conclusive results became. Secondly, results for quality of reviews were more diverse, but a trend towards higher quality reviews coming to a positive conclusion can be found. Therefore one could assume that acupuncture might be effective for FMS.

For nutritional supplement one review from 2003 [52] found positive results while one review from 2011 [53] found negative evidence. One could assume that nutritional supplements are not effective in the treatment of FMS.

Results of the overview are mostly in line with a previous overview [45]; however there were also substantial differences. While Terry et al. [45] only included 5 reviews, we could include 25. Results on hydrotherapy are positive in both overviews; the results for massage however are more diverse, which might have been a result of the inclusion of massage into the category of manual therapies. According to Terry et al. [45] also the use of acupuncture appears promising. In our review we also found that especially high quality reviews supported positive effects of acupuncture, and this finding might however be influenced by other factors such as time which might be connected to better methodological training or more eligible trials. For homeopathy on the other hand the quality of evidence was not supportive of positive or promising results yet.

The evidence found in this review is not necessarily in line with current treatment guidelines; for example, there is conflicting or lacking evidence for qigong, tai chi, or yoga; nevertheless they are explicitly recommended in the German guideline [75] because physical activity and self-care are considered important therapeutic goals in fibromyalgia patients. Positive evidence on massage on the other hand did not lead to a positive recommendation per se; instead availability, costs, and its negative influence on self-efficacy and coping behavior led to downgrading of recommendation.

### 4.3. Limitations

Despite all previous findings and conclusion, there are several limitations that might limit the results of this overview.

The first limitation concerns the inclusion criteria of the included reviews. While some reviews explicitly included only certain CAM therapies, others conducted comprehensive reviews for CAM in general. Since the definition of CAM is not always consistent, this might lead to a different direction of each review. Two reviews aiming at the efficacy of CAM therapies in general might therefore end up in contrary conclusions merely by different definitions of CAM. Also reviews that focused on physiotherapeutic interventions or exercise in general might also have summarized evidence for single CAM interventions; however since their focus was not on CAM only, they were not included. Furthermore reviews and the trials they were based on might have used different diagnostic criteria or procedures, which also might have influenced the results.

The methodological quality of reviews is probably one of the major limitations. Quality ranged from lowest to highest possible, and quality of reviews was also associated with the direction of results. Particularly for topics with only low quality reviews available this might limit the validity of results. This might be problematic since reviews and meta-analysis are considered highest evidence standard for guideline developers.

And another limitation concerns the use of terminology when summarizing evidence, risk of bias, methodological quality, or recommendations. Most reviews used different terms that were not consistent with recommendations; this might result in misinterpretation of results and conclusions.

### 4.4. Implication for Further Research

In the future the number of reviews and meta-analyses will increase; this should be accompanied by increased methodological quality, if those reviews aim to influence treatment guidelines and decision makers. The more transparent and methodologically sound a review is conducted the easier it is for decision makers to rely on the results when developing treatment guidelines. The following implications, which are not limited to CAM reviews, can be made for future reviews.

The basis of a good review is a straight and relevant research question. Defining patients, interventions, comparators, outcomes, and study types before conducting a review are most essential. They are also the basis of a comprehensive literature search. All steps from literature search to conclusions must be made visible; this also includes information such as the following. Was the search limited in terms of language or publication status? Who extracted data? Why were studies excluded? What studies were finally included? How was the quality of those trials? How were results combined, for example, using a meta-analysis? How did one come up with a conclusion or recommendation? There are several guidelines available on how to report a systematic review; for example, the PRISMA statement [10] provides a comprehensive list of items to be reported in reviews and meta-analyses. There are also validated tools for risk of bias or quality assessment, for example, Cochrane tools, and for grading recommendations, for example, GRADE [76]. Since those tools are validated as a whole, they should not be altered. For several therapies such as massages blinding of patients and therapists is impossible; however this per se constitutes a possible risk of bias in those trials, which cannot be eliminated by eliminating that item from the risk of bias assessment list.

In summary, the number of reviews on CAM therapies for the management of fibromyalgia syndrome is increasing. However many reviews still show certain methodological flaws limiting definite conclusions about the efficacy of CAM therapies. Rather consistent positive results were found for tai chi, yoga, meditation- and mindfulness-based interventions, hypnosis or guided imagery, EMG biofeedback, and balneotherapy/hydrotherapy. Inconsistent results concerned qigong, acupuncture, chiropractic interventions, EEG biofeedback, and nutritional supplements. Inconclusive results were found for homeopathy and phytotherapy.

## 5. Conclusion

Despite a growing body of scientific evidence of CAM therapies for the management of FMS systematic reviews still show methodological flaws limiting definite conclusions about the efficacy and safety of those therapies.

## Figures and Tables

**Table 1:** Characteristics of included systematic reviews.

Reference	Year	Meta-analysis	Intervention	Comparator groups	Types of included studies	Number of studies Number of patients	Risk of bias assessment? Grade of Recommendation?	Safety (AE/SAE)
CAM interventions in general								
Baranowsky et al. [50]	2009	No	CAM therapies as defined by the NIH, with the exception of dietary, nutritional, herbal, and hormonal supplements	Any	RCT	24; 1466	Adapted instrument	Not assessed; not reported
De Silva et al. [51]	2010	No	CAM therapies (oral or topical application)	Placebo or other treatments	RCT	7; 267	Jadad scale	Minor AE No SAE
Holdcraft et al. [52]	2003	No	CAM therapies according to NIH scheme	Any	RCT, non-RCT	22; 1091	Consort rating system	Not assessed; not reported
Terhorst et al. [53]	2011	Yes	CAM therapies according to NIH scheme	Any	RCTs	60; 2897	Adapted GRADE system (random sequence generation and allocation concealment; incomplete outcome data; selective reporting; other)	Not assessed; not reported

Exercise-based CAM								
Chan et al. [54]	2012	No	Qigong	Any	RCT, CT	4; 251	Jadad scale	Not assessed; not reported
Langhorst et al. [55]	2013	Yes	Meditative movement therapies (yoga, qigong, tai chi)	Any control	RCT	7; 362	Randomization, allocation concealment, blinding of outcome assessment, adequacy of data analysis (intention-to-treat analysis)	3.1% AE No SAE
Lauche et al. [56]	2013	Yes	Qigong	Any control	RCT	7; 395	Cochrane risk of bias assessment; GRADE	Minor AE No SAE
Mist et al. [57]	2013	Yes	Complementary and alternative exercise	Any	Any	16; 832	Jadad score	No SAE

Manipulative therapies								
Ernst [58]	2009	No	Chiropractic treatment	Any	RCT	4; 108	Jadad score	Not assessed; not reported
Kalichman [59]	2010	No	Massage therapy	Any	Any, with an emphasis on RCTs	8; 235	None	Not assessed; not reported
Schneider et al. [60]	2009	No	Chiropractic treatment	Any	RCTs, systematic reviews, meta-analyses, guidelines consensus documents, RCTs, clinical trials, case reports, case-control studies, surveys	8 systematic reviews, 3 meta-analyses, 5 practice guidelines, 44 RCTs, 17 clinical trials, 17 case reports, 2 case-control studies, 2 surveys	Oxford rating scale; Scottish Intercollegiate Guidelines Network evidence rating checklist	Not assessed; not reported

Mind/Body interventions								
Bernardy et al. [61]	2011	Yes	Hypnosis, guided imagery	Any	CT; quasi-RCT; RCT	6; 239	Scale for rating the quality of psychological trials in pain [40]	Minor AE No SAE
Glombiewski et al. [62]	2013	Yes	EMG and EEG biofeedback	Any	RCT	7; 321	Randomization, allocation concealment, blinding of outcome assessor, adequate data analysis, intention-to-treat analysis	Minor AE No SAE
Hadhazy et al. [63]	2000	No	Mind-Body therapies (education, cognitive therapy, movement therapy, dance, meditation, relaxation, hypnosis, guided imagery, biofeedback)	Any	RCT, quasi-RCT	13; 802	Jadad criteria	No AE reported
Kozasa et al. [64]	2012	No	Meditation-based interventions	Any	RCT	4; 449	Jadad score	Not assessed; not reported
Lauche et al. [65]	2013	Yes	Mindfulness-based stress reduction (MBSR)	Any control	CT; RCT	6; 674	Cochrane risk of bias assessment; GRADE	No AE/SAE reported

Acupuncture								
Deare et al. [66]	2013	Yes	Acupuncture (invasive acupuncture only) as standalone or adjunct	Any (nonacupuncture treatments, placebo, sham-acupuncture)	RCT	9; 395	Cochrane risk of bias assessment	0–53% AE No SAE
Langhorst et al. [67]	2010	Yes	Acupuncture	Sham-acupuncture, simulated acupuncture	RCT, quasi-RCT	7; 385	van Tulder score	3–70% AE No SAE
Martin-Sanchez et al. [68]	2009	Yes	Acupuncture	Sham-acupuncture	RCT	6; 323	None	Not assessed; not reported
Mayhew and Ernst [69]	2007	No	Acupuncture	Any	RCT	5; 316	Jadad score	Not assessed; not reported

Balneotherapy/hydrotherapy								
Fraioli et al. [70]	2013	No	Spa therapy	Unclear	RCTs, observational studies	7; 303	Value of the international journals that published these researches, the number of patients included in the studies, the methods used to study the patients, and the possibility of exclusion of more frequent studies bias	Not assessed; not reported
Langhorst et al. [71]	2009	Yes	Hydrotherapy	Any	RCT	13; 446	van Tulder score	Not assessed; not reported
McVeigh et al. [72]	2008	No	Hydrotherapy	Any	RCT	10; 571	van Tulder score	Not assessed; not reported

Phytotherapy								
de Souza Nascimento et al. [73]	2013	No	Medicinal plants or related natural products	Placebo, other drugs	RCTs	8; 475	Jadad score	Minor AE No SAE

Homeopathy								
Perry et al. [74]	2010	No	Homeopathy	Any	RCTs	4; 163	Jadad score; recommendations from the Cochrane Handbook	Not assessed; not reported

AE: adverse event; CAM: complementary and alternative medicine; CONSORT: Consolidated Standards of Reporting Trials; CT: controlled trial; GRADE: Grading of Recommendations Assessment, Development, and Evaluation; RCT: randomized controlled trial; SAE: serious adverse event.

**Table 2:** AMSTAR rating table of included systematic reviews.

Reference	Year	A priori design?	Two data extractor and consensus?	Comprehensive literature search?	Statement on inclusion of grey literature? Language?	List of included and excluded studies?	Characteristics of studies provided, for example, tables?	Quality of risk of bias assessment?	Scientific quality of the included studies used appropriately in formulating conclusions?	Methods used to combine the findings of studies appropriate? Test on heterogeneity	Likelihood of publication bias assessed?	Conflict of interests stated?	Sum
CAM in general													
Baranowsky et al. [50]	2009	Yes	Cannot answer	Yes	No	No	Yes	Yes	No	Not applicable	Not applicable	No	4
De Silva et al. [51]	2010	Yes	No	Yes	No	No	No	Yes	Yes	Not applicable	Not applicable	Yes	5
Holdcraft et al. [52]	2003	Yes	Cannot answer	Yes	No	No	No	Yes	Yes	Not applicable	Not applicable	No	4
Terhorst et al. [53]	2011	Yes	No	Yes	Yes	No	No	Yes	No	Yes	Yes	Yes	6

Exercise-based CAM													
Chan et al. [54]	2012	Yes	No	Yes	No	No	Yes	Yes	Yes	Not applicable	Not applicable	Yes	6
Langhorst et al. [55]	2013	Yes	Yes	Yes	No	Yes	Yes	Yes	No	Yes	Yes	Yes	9
Lauche et al. [56]	2013	Yes	Yes	Yes	No	Yes	Yes	Yes	Yes	Yes	Yes	Yes	10
Mist et al. [57]	2013	Yes	Cannot answer	Yes	No	No	Yes	Yes	No	No	Yes	Yes	6

Manipulative therapies													
Ernst [58]	2009	Yes	Yes	Yes	No	No	No	Yes	Yes	Not applicable	Not applicable	Yes	6
Kalichman [59]	2010	Cannot answer	Cannot answer	Yes	No	No	Yes	No	No	Not applicable	Not applicable	Yes	3
Schneider et al. [60]	2009	Yes	Cannot answer	Yes	Yes	No	No	Yes	No	Not applicable	Not applicable	Yes	5

Mind/Body interventions													
Bernardy et al. [61]	2011	Yes	Yes	Yes	Yes	Yes	Yes	Yes	Yes	Yes	Yes	Yes	11
Glombiewski et al. [62]	2013	Yes	Yes	Yes	Yes	Yes	Yes	Yes	No	Yes	Yes	Yes	10
Hadhazy et al. [63]	2000	Yes	Yes	Yes	Yes	No	Yes	Yes	Yes	Not applicable	Not applicable	No	7
Kozasa et al. [64]	2012	Cannot answer	Cannot answer	No	No	No	No	Yes	No	Not applicable	Not applicable	Yes	2
Lauche et al. [65]	2013	Yes	Yes	Yes	No	Yes	Yes	Yes	Yes	Yes	Yes	Yes	10

Acupuncture													
Deare et al. [66]	2013	Yes	Yes	Yes	Yes	Yes	Yes	Yes	Yes	Yes	No	Yes	11
Langhorst et al. [67]	2010	Yes	Yes	Yes	Yes	No	Yes	Yes	Yes	Yes	No	Yes	9
Martin-Sanchez et al. [68]	2009	Cannot answer	Cannot answer	Yes	No	No	No	No	No	Yes	No	Yes	3
Mayhew and Ernst [69]	2007	Yes	Yes	Yes	No	No	No	Yes	No	Not applicable	Not applicable	Yes	5

Balneotherapy/hydrotherapy													
Fraioli et al. [70]	2013	Cannot answer	Cannot answer	Yes	No	No	No	No	No	Not applicable	Not applicable	Yes	2
Langhorst et al. [71]	2009	Yes	Yes	Yes	Yes	Yes	Yes	Yes	Yes	Yes	Yes	Yes	11
McVeigh et al. [72]	2008	Yes	Yes	Yes	No	Yes	No	Yes	Yes	Not applicable	Not applicable	No	6

Phytotherapy													
de Souza Nascimento et al. [73]	2013	Yes	Yes	Yes	No	No	Yes	Yes	Yes	Not applicable	Not applicable	Yes	7

Homeopathy													
Perry et al. [74]	2010	Yes	Yes	Yes	No	No	No	Yes	Yes	Not applicable	Not applicable	Yes	5

**Table 3:** Overview of conclusions for investigated therapies from included systematic reviews.

Intervention	Positive evidence	Negative evidence	Inconclusive
Mind/Body interventions			
Mind/Body interventions in general	(i) Effects on pain [53] (ii) More effective than usual care for some outcomes [63]		
Meditation-based interventions	(i) Mostly positive results [50] (ii) Most studies indicate improvement [64]		
Mindfulness-based stress reduction	Moderate short-term effects on FMS key symptoms [65]		
Hypnosis/guided imagery	Strong short-term effects on pain [61]		
Biofeedback	(i) Limited evidence for biofeedback [52] (ii) Moderate effects on pain for EMG biofeedback [62]	(i) No positive results [50] (ii) No effects of EEG biofeedback [62]	
Relaxation	Limited evidence [52]		
Autogenic training		No effects of autogenic training [50]	

Exercised-based CAM			
Qigong	(i) Moderate-to-strong short-term effects on FMS key symptoms [56] (ii) Moderate effect on functional disability [57] (iii) A positive trend [53]	(i) No positive results [50] (ii) No evidence of effects [55]	Too early to draw conclusions [54]
Tai Chi	(i) Strong effect on functional disability [57] (ii) Moderate effect on sleep quality [55] (iii) One study in favor of tai chi [53]		
Yoga	(i) Significant effects on pain, fatigue, depression, and quality of life [55] (ii) Moderate effect on functional disability [57]		

Manipulative therapies			
Chiropractic interventions	Limited evidence [60]	(i) No positive evidence [58] (ii) Insufficient evidence for benefit [52]	Not enough evidence [53]
Massage	(i) Moderate evidence [52] (ii) Massage is beneficial for patients with fibromyalgia [59]	Ineffective [53]	

Acupuncture			
	(i) Strong evidence [52] (ii) Strong evidence for small short-term effects on pain [67] (iii) Low-to-moderate evidence for acupuncture compared to usual care or standard care [66]	(i) Ineffective [53] (ii) No evidence for effectiveness [68]	(i) Mixed quality [50] (ii) Effectiveness not yet supported, mixed evidence [69]

Balneotherapy/hydrotherapy			
	(i) Positive evidence [50] (ii) Limited evidence [52] (iii) Effects on pain [53] (iv) Moderate evidence for pain [69] (v) Strong evidence [72] (vi) Appears efficacious [70]		

Phytotherapy			
			Unclear whether medicinal products or related natural products are effective [73]

Nutritional supplements			
	Limited evidence for diverse supplements [52]	Ineffective [53]	

Homeopathy			
	(i) Positive results [50] (ii) Limited evidence [52] (iii) Some evidence [51]		(i) Not enough evidence [53] (ii) Effectiveness remains unproven [74]
